# Inhibition of microvesiculation sensitizes prostate cancer cells to chemotherapy and reduces docetaxel dose required to limit tumor growth *in vivo*

**DOI:** 10.1038/srep13006

**Published:** 2015-08-25

**Authors:** Samireh Jorfi, Ephraim A. Ansa-Addo, Sharad Kholia, Dan Stratton, Shaunelle Valley, Sigrun Lange, Jameel Inal

**Affiliations:** 1 Cellular and Molecular Immunology Research Centre, School of Human Sciences, London Metropolitan University, U.K; 2 University College London School of Pharmacy, 29-39 Brunswick Square, London WC1N 1AX, U.K

## Abstract

Microvesicles shed from cells carry constituents of the cell cytoplasm, including, of importance in multidrug resistance to cancer chemotherapy, drugs that the tumor cell attempts to efflux. To see whether such drugs could be used at lower concentrations with the same efficacy, it was first shown that microvesiculation of prostate cancer (PCa) cells, PC3, could be inhibited pharmacologically with calpeptin (calpain inhibitor) and by siRNA (*CAPNS1*). In cells treated with docetaxel (DTX), this inhibition resulted in a third-fold increase in intracellular concentrations of DTX. As a result, 20-fold lower concentrations of DTX (5 nM) could be used, in the presence of calpeptin (20 μM) inducing the same degree of apoptosis after 48 h in PC3 cells, as 100 nM of DTX alone. Inhibition of microvesiculation similarly improved combination chemotherapy (DTX and methotrexate). In a mouse xenograft model of PCa, DTX (0.1 mg/kg) together with calpeptin (10 mg/kg), administered i.p., significantly reduced tumor volumes compared to DTX alone (0.1 mg/kg) and brought about the same reductions in tumor growth as 10 mg/kg of DTX alone. As well as further reducing vascularization, it also increased apoptosis and reduced proliferation of PC3 cells in tumor xenografts.

Multidrug resistance (MDR) in solid tumors of which there are two types, intrinsic, present before treatment, and acquired which develops during treatment^1^ remains an unresolved problem in cancer chemotherapy. To compound the problem, multiple pathways are affected in MDR, acting synergistically to develop resistance^1^.

The ABC transporter superfamily members are involved in drug efflux. P glycoprotein, P-gp was the first protein found to confer drug resistance on cultured cells^2^ and the Multidrug-Resistance-associated Protein (MRP1) is involved in the resistance of lung cancer cells^3^. P-gp inhibitors such as cyclosporine and valspodar, however, are promiscuous in terms of transporter specificity^4^. Although P-gp is not expressed in PCa cell lines, it was instrumental in proving a non-genetic mechanism involved in multidrug resistance by co-culturing P-gp positive chemo-resistant leukemic cells with P-gp negative, chemo-sensitive cells, resulting in direct cell-cell transfer and expression of P-gp^5^. Others went further to elucidate the mechanism, showing microvesicle- (MV-) mediated transfer^6^. Although docetaxel (DTX) is used in the treatment of metastatic castrate-resistant PCa, many patients do not respond to DTX therapy and all will inevitably develop resistance^7^. Therefore the understanding of the mechanisms by which resistance develops is necessary to develop novel therapies.

In practise, drug resistance occurs due to changes in the rate of drug uptake and efflux, altered drug metabolism, decreased drug-target complex formation or enhanced DNA repair mechanisms^8^. More specific examples include decreased expression of reduced folate carrier (RFC) associated with methotrexate (MTX) resistance^2^, and resistance to DTX resulting from mutation at the drug binding site of tubulin and the expression of different tubulin isoforms^9^. In this study, however, we focused our attention on the removal of chemotherapeutic drugs from cancer cells through the release of MVs.

Microvesicles are small plasma membrane-derived vesicles present in blood, urine and other body fluids that carry cytosolic and plasma membrane constituents from the parent cells, including bioactive molecules, proteins, mRNAs and miRNAs; their roles in diagnosis, prognosis and in surveillance of diseases have become increasingly apparent in recent times^10^. Cancer cells release MVs to protect themselves against intracellular stress and inhibition of this release leads to accumulation of caspase-3 and cell apoptosis^11^. MVs released from cancer cells containing chemotherapeutic agents, aid drug resistance and interact with the immune system to suppress immune response to tumors^12^. Cancer cells may also hide from the immune system by fusing with healthy MVs, expressing their membrane-specific proteins^13^ and may go on to aid cancer progression through the metalloproteases they carry, facilitating extracellular matrix degradation^14^,^15^. In terms of drug therapy and MVs we became interested in increased efflux of anticancer drugs^16^,^17^ mediated by MVs as a mechanism for multidrug resistance.

DTX binds β-tubulin monomers. By thus stabilizing microtubules and preventing their depolymerization, microtubular dynamics is effectively arrested which impacts on a host of essential cellular functions. Inhibition of mitotic cell division blocks cells in the G2/M phase, thus inhibiting cell proliferation. DTX also inhibits early endosomal uptake and secretion. MTX, which we also used in this study, however, is an antimetabolite and antifolate drug, that inhibits DNA, RNA and protein synthesis.

Calpains comprise a family of calcium-dependent intracellular cysteine proteases involved in processes important for tumor progression including apoptosis, proliferation, cell migration and invasiveness. Most calpain activity is due to μ- and m- calpains (or Calpain-1 (CAPN1) and Calpain-2 (CAPN2), respectively); these calpains in turn require μM and mM concentrations of calcium to function. They are heterodimers comprising a large 80 kDa catalytic subunit and a shared 28 kDa regulatory subunit, CAPNS1 (calpain small-1) (sometimes called Calpain-4). In tumor cells calpain is usually hyperactivated and due to its ability to cleave cytoskeleton is involved in tumor migration and metastasis. Calpeptin is therefore a cell-permeable protease inhibitor which selectively inhibits calpains and reduces MV release^18^,^19^,^20^.

We believed drug-detoxification by cancer cells based on MV release could explain the cells’ insensitivity to drug-induced apoptosis and so the aim of this study was to improve cancer chemotherapy and circumvent drug resistance through regulation of MV release. By knocking down CAPNS1 expression with siRNA or pharmacologically inhibiting it with calpeptin, we wanted to see if this could sensitize cancer cells to chemotherapy allowing a reduction in the effective drug dosage.

## Methods

### Ethical statement

All experimental protocols in this study were carried out in accordance with approved guidelines (Faculty safety code of practice) and all experimental protocols were approved by the Faculty of Life Sciences (FLS), including the FLS London Metropolitan University Research Ethics Review panel and Genetic Modifications Safety Committee. The intraperitoneal (i.p.) xenograft model of human PCa was carried out at Washington Biotechnology (Baltimore, MD), (U.S. Department of Agriculture (USDA) Registration No. 51-R-031 and Accreditation No. A4192-01) where animal care was according to the Public Health Services guideline, and in accordance with the Office for Laboratory and Animal Welfare division of the National Institutes of Health.

### Knockdown of CAPNS1 by small interfering RNA (siRNA) transfection

GeneSolution siRNA sequences targeted to four different sites in calpain small subunit 1, *CAPNS1* mRNA (GenBank Accession No. X04106) and negative control siRNA (Qiagen, Crawley, UK) were reconstituted in sterile RNase-free water at a final concentration of 10 μM. PC3 cells (5 × 10^4^/well in triplicate) were transfected with 5 or 50 nM siRNA (final concentration), using HiPerfect transfection reagent (HPP, Qiagen) for 48 h prior to performing experiments. The sequences for the human *CAPNS1* siRNAs are indicated in Supplementary Fig. 1. Consistent reduction of CAPNS1 expression was observed with siRNA#6 which was used to assess the effects of decreasing CAPNS1 levels on the sensitivity of PC3 cells to drug resistance.

### Immunoblotting analysis of siRNA transfected cells

Control or CAPNS1 knocked down PC3 cells, were treated with lysis buffer (100 mM HEPES/KOH, 2 mM CaCl_2_, 0.5% Triton X-100) containing a protease inhibitor cocktail (Sigma-Aldrich). Protein lysate concentrations were measured using the BCA assay kit (Pierce Biosciences)^21^ and 20 μg resolved by SDS-PAGE on a 12% polyacrylamide gel^21^. Immunoblotting was carried out as described before^21^, this time being incubated with anti-β-actin or anti-CAPNS1 (*CAPNS1*) (both Sigma-Aldrich), diluted 1/500 in PBST (PBS with 0.1% (v/v) Tween 20). After incubating with secondary antibodies, protein bands were visualised using the LumiGOLD ECL Western Blotting Detection kit (SignaGen Laboratories), the chemiluminescence signal being detected on the ChemiDoc-It Imaging System (UVP, Cambridge).

### Cell culture and proliferation assay

Adherent PC3 cells were maintained at 37 °C with 5% CO_2_, in RPMI growth medium, and split, depending on confluency, every 3 to 5 days. The number of cells and viability were determined before the start of every experiment using the flow cytometer (ViaCount assay, Guava Technologies), exponentially growing cells with viability of 95% or higher being routinely used. To measure proliferation and any inhibitory effect of CP in combination with DTX treatment, PC3 cells were seeded at 5 × 10^4^ cells per well in 6-well plates and 24 h later given 5 nM DTX with or without CP (20 μM), control cells being given DMSO (0.01%). Cells at 1, 2 and 3 day time points were detached with trypsin and after trypan blue staining (0.4%) counted on a haemocytometer.

### Isolation and analysis of microvesicles from conditioned medium

Isolation and analysis of MVs, which included enumeration by nanosight tracking analysis (NTA) detection of phosphatidylserine (PS) by flow cytometry (Guava EasyCyte, Guava Technologies) and the use of electron microscopy to confirm size and purity was essentially carried out as described before^21^,^22^,^23^,^24^,^25^,^26^,^27^ with some modifications.

To purify MVs, cell culture supernatants were centrifuged at 200 *g* for 5 min to remove cells, 4,000 *g* for 1 h to remove cell debris and then at 15,000 *g* for 2 h to pellet MVs. After washing in exosome and MV-(EMV-) free, sterile PBS, the pellet was resuspended in EMV-free PBS and quantified by nanosight tracking analysis (NTA). The nanosight used to enumerate MVs was the NS500 (Nanosight, Amesbury, UK), equipped with a sCMOS camera and a 405 nm diode laser. Data acquisition and processing were performed using NTA software 3.0. Background extraction and automatic settings were applied for the minimum expected particle size, minimum track length and blur, the ambient temperature being set at 23 °C. Silica beads (100 nm diameter; Microspheres-Nanospheres, Cold Spring, NY) were used to calibrate the NS500. Samples were diluted 10–50 fold in EMV-free PBS to maintain the number of particles in the field of view between approximately 20–40. For each sample, 4 × 30 s videos were recorded, replicate histograms being averaged. Analysis was only carried out on measurements with at least 1000 completed tracks.

### DTX- and MTX-mediated apoptosis of PC3 cells

PC3 cells seeded at 5 × 10^4^/well in triplicate were washed after 24 h and treated with calpeptin (CP) (20 μM for 45 min), re-washed and resuspended in varying concentrations of MTX and DTX for 48 h. DTX/MTX-induced apoptosis levels in the presence or absence of CP were assayed using Guava ViaCount by flow cytometry.

### Drug extraction from MVs and HPLC

The MV samples were extracted in a solution of 9 parts dichloromethane: 1 part propan-2-ol with gentle mixing. Following protein precipitation (10% TCA) and centrifugation the supernatant was removed and 20 μl used for multistep gradient HPLC using a C_18_ column with UltiMate 3000 variable-wavelength detector. The mobile phase of 0.5% H_3_PO_4_/acetonitrile was pumped at 1 ml/min. The UV detector was set at 254 nm for a total run time of 23 min alternating flow between acetonitrile and phosphoric acid. As the system uses an automated sampler, all pre-made samples and MTX standards 3.06, 6.125, 12.25, 50 and 100 μM, were run on the system in duplicate at a sequence time of 12 min and peaks observed at UV Vis 302 nm. With the retention time for MTX established at 12.5 min, the Chromeleon software of the Dionex D3 system was used to produce specific high resolution chromatographs of the drugs.

### Docetaxel uptake in PC3 cells

PC3 cells were attached at 1 × 10^5^ cells per well in 6-well plates over 24 h. Cells were then treated with CP (20 μM) and DTX (100 nM) and after 2 h, cells were washed four times and lysed (0.7% NP40; Tris.Cl, pH 7.4; 70 mM EDTA; 200 nM NaCl on ice for 10 min). After protein quantitation, (BCA assay) the sample was extracted with acetonitrile and the supernatants (15,000 *g*; 15 min) analysed by HPLC to determine DTX concentration, the result being normalised to nmol DTX per mg of cell protein.

### Xenograft model of human prostate cancer

Male, 6–8 week old athymic nude mice (*nu/nu*) (8 mice per group) were injected i.p. in each flank with 5 × 10^6^ PC3 cells (suspended in 100 μl PBS). After about 7 days, once the tumors became palpable, usually reaching an average volume of 100 mm^3^, weekly therapy was started, tumor growth being monitored 3 times weekly using calipers to estimate tumor volume from the formula V = (length × width × height) × 0.52. Besides the drugs DTX and MTX, alone, or with calpeptin (CP) injected at 10 mg/kg, 1 h before drugs, mice were also treated weekly by intratumoral (i.t.) injection of 10 μg siRNA with 7 μl Oligofectamine^TM^ (Invitrogen). Throughout, mice were weighed twice weekly. Mice were sacrificed before reaching the maximum tumor size of 2000 mm^3^. At necropsy, excised tumors were weighed, fixed in 10% buffered formalin, and embedded in paraffin for immunohistochemical analysis. Blood samples were collected on 200 μl of 0.13 M sodium citrate and MVs isolated as described before^24^, labeled with AnV-FITC for 15 min at RT and then diluted in 500 μl binding buffer. Samples were analysed by nanosight tracking analysis as described above.

### Immunohistochemical analysis of resected xenografts

Before immunohistochemical analysis, the 5 μm tissue sections were deparaffinized in xylene and dehydrated as described before^28^. Antigen retrieval for anti-Ki-67 staining required boiling in 5 mM citrate buffer for 15 min. To block non-specific sites, samples were incubated for 1 h at RT with 5% BSA. All antibodies (rat anti-mouse CD31 (PECAM-1), BD, Pharmingen; rabbit monoclonal anti-human Ki-67 (1:200), DAKO, Denmark) were diluted in 10 mM Tris-HCl buffer (pH 7.8) and 1.0% BSA (w/v). After incubation with primary antibody, tissue sections were given three 10 min washes in Tris buffer, and incubated with HRP-conjugated anti-rabbit or mouse IgG (1:100, DakoCytomation) for 2 h at RT. Peroxidase activity was developed using diaminobenzidine^28^. The tissue sections were counter-stained with haematoxylin and negative controls were stained without primary antibody or with pre-immune serum. For each section, eight random fields were scored independently by two technicians in a blinded manner. Processing of resected tumors for this research was carried out according to the standard guidelines of the School of Human Sciences Ethics Committee.

### *In vivo* detection of apoptosis via TUNEL assay

To detect apoptotic cells in resected tumors, terminal deoxynucleotidyl transferase-mediated dUTP nick-end labeling (TUNEL) staining was carried out using the TdT *In Situ* Apoptosis Detection Kit (R&D Systems) according to the manufacturer’s instructions. Light microscopy was used to calculate the percentage of apoptotic (TUNEL-positive cells).

### Statistical analysis

Data are presented as the mean ± S.E.M. for each experimental group, the differences between these groups being analyzed by one- or two-way analysis of the variance (ANOVA). To determine any significance in difference of the tumor volumes between control and the various treatment groups, the non-parametric Mann-Whitney U test was used. One-way ANOVA followed by the Bonferroni multiple comparison test was also carried out using GraphPad Prism 6 to assess inter-group differences. *P* values were two-sided (unless otherwise stated) and differences were considered significantly different at: *p < 0.05; **p < 0.01; ***p < 0.005.

## Results

### MVs released from MTX-treated cells carry MTX

**Figure 1:**
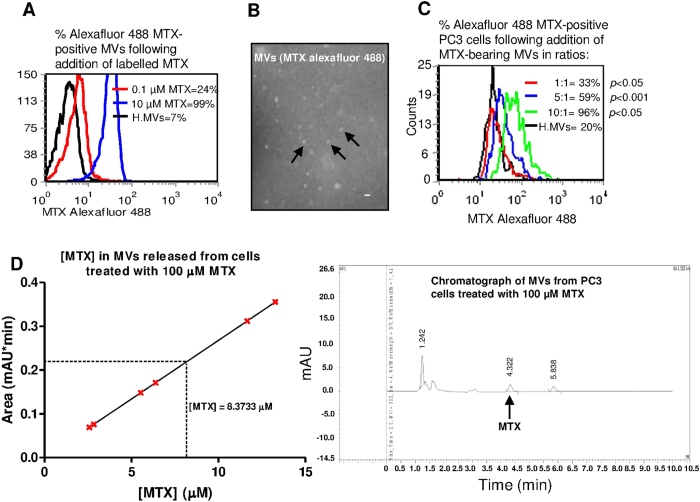
Labeled MTX in MVs released from PC3 cells transfer fluorescent drug to recipient cells. PC3 cells (5 × 10^4^/well) were treated with 10 μM labeled MTX Alexafluor 488. MVs from these cells were shown to carry fluorescent MTX (**A**,**B**) (where Bar is 500 nm at X60 magnification using a 1 × 81 motorized, inverted fluorescence microscope; Olympus). Upon addition to healthy, recipient PC3 cells (5 × 10^5^/well) in different ratios drug transfer was demonstrated (**C**). The concentration of MTX in MVs released from cells treated with 100 μM MTX was shown by HPLC to be 8.4 μM; the standard curve and chromatograph of MTX-bearing MVs is shown in (**D**) (and associated chromatographs in Figs S2 and S3).

The concept that MVs shed cytosolic components from cells prompted us to look for chemotherapeutic drug in MVs released from PC3 cells placed in growth medium with MTX AlexaFluor 488 (Lab MTX) (10 μM; 1 h). The isolated MVs showed 99% relative MTX Alexafluor fluorescence, by flow cytometry, compared to ‘healthy’ (H.MVs) isolated from untreated cells (Fig. 1A) as well as positive fluorescence by microscopy, Fig. 1B. PC3 cells when co-cultured with MTX-bearing MVs, isolated from PC3 cells treated with Lab MTX, were found to receive a significant transfer of fluorescence, in a dose-dependent manner (Fig. 1C).

We also showed by HPLC that PC3 cells treated with high dose MTX (100 μM) release MTX-bearing MVs, and were able to establish an average concentration within these MVs of 8.4 μM MTX (Fig. 1D and Supplementary Figs 2 and 3).

### Downregulation of calpain expression with siRNA decreased MV release from PC3 cells and increases drug-induced apoptosis in PC3 cells

**Figure 2:**
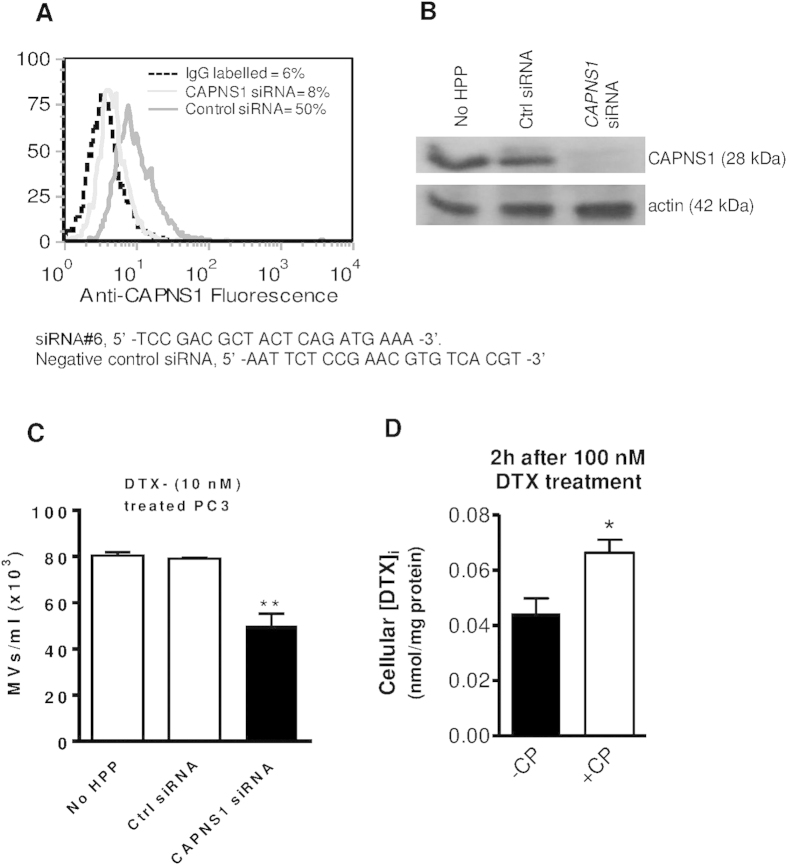
Silencing *CAPNS1* in PC3 cells reduces DTX-stimulated MV release and pharmacological inhibition of calpain increases cellular concentrations of DTX. PC3 cells were transfected with CAPNS1 siRNA#6 (5 and 50 nM) and incubated at 37 °C/5% CO_2_ for 48 h. Decreased CAPNS1 expression was shown by flow cytometry (**A**) and Western blotting (**B**) resulting in cells with a reduced capacity for MV release (**C**). Intracellular DTX in PC3 cells was assayed by HPLC following DTX treatment and showed an increase when microvesiculation was inhibited, by pretreatment with CP (20 μM) (**D**).

**Figure 3:**
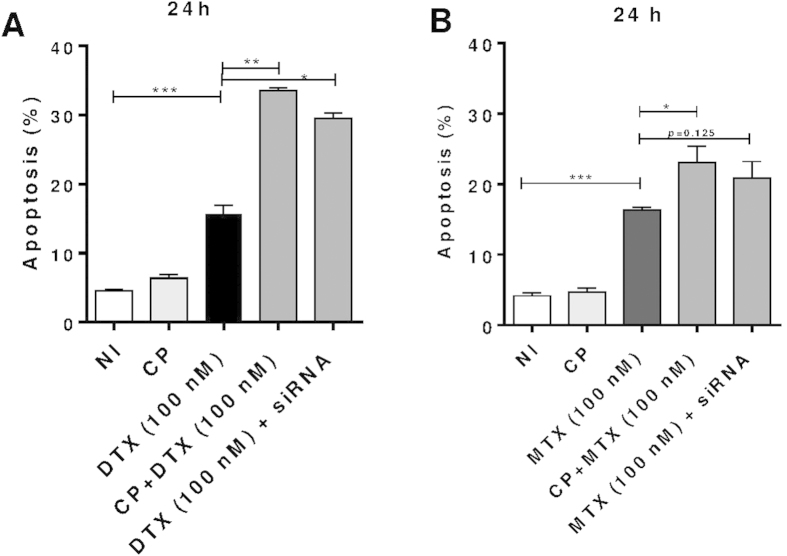
Apoptosis levels induced with DTX or MTX are significantly increased upon silencing CAPNS1 in PC3 cells. Silencing calpain in PC3 cells significantly increased apoptosis levels induced with DTX (**A**) or MTX (**B**) after 24 h. *CAPNS1* silenced PC3 cells (transfected with siRNA#6) were washed and treated with DTX or MTX (100 nM) for 24 h and apoptosis levels assessed using Viacount. Control CP-treated cells were given 20 μM CP.

To further investigate the role of calpain in MV release, we carried out a knockdown of both μ- and m-calpain isoforms using calpain small-subunit 1 small interfering RNA (CAPNSI siRNA). Firstly, PC3 cells were transfected with CAPNS1 siRNA. After fixation and permeabilisation, anti-CAPNS1 labelled cells were analysed using the Express plus assay by flow cytometry, Fig. 2A, and by Western blotting, Fig. 2B, which confirmed effective silencing. In addition to pharmacological reduction of microvesiculation, using CP, (Supplementary Fig. S4a) therefore, knockdown of CAPNS1 expression significantly reduced MV release from DTX-treated PC3 cells (Fig. 2C), resulting in a one third elevation of the intracellular concentration of DTX as determined by HPLC assay (Fig. 2D). Furthermore, PC3 cells silenced for CAPNS1 when treated with DTX (Fig. 3A) and MTX (Fig. 3B) at 100 nM for just 24 h already showed increased susceptibility to chemotherapy compared to control cells, although a non-significant increase was obtained with 5 nM siRNA in conjunction with MTX (Fig. 3B).

### Inhibition of calpain elevates drug-induced apoptosis of prostate cancer cells PC3, LnCaP and Du-145 cells and further limits proliferation of PC3

We postulated that MV release from cancer cells during chemotherapy expels drugs thereby facilitating cell survival and that if MV release could be inhibited (initially using CP), lower concentrations of chemotherapeutic drug could still achieve significant levels of apoptosis.

**Figure 4:**
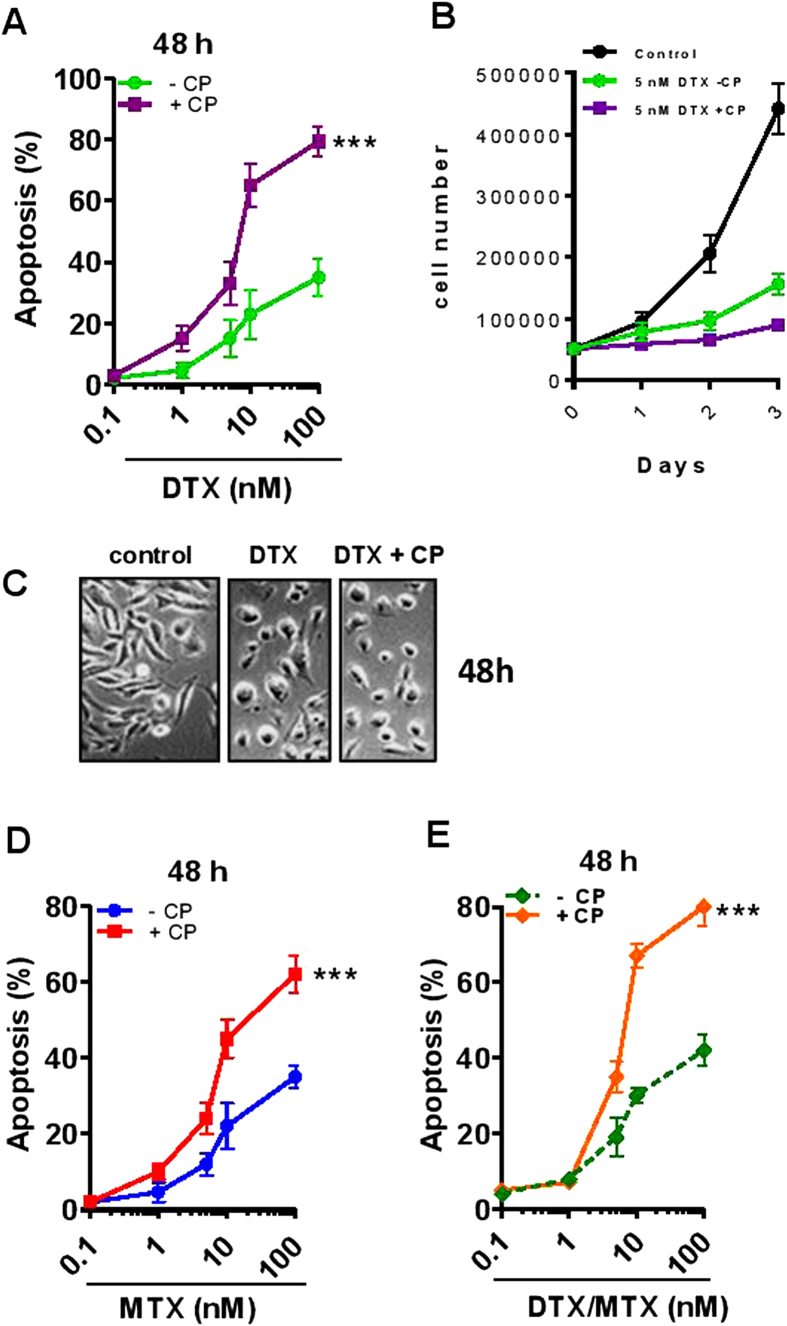
DTX- and MTX-treated PCa cells undergo significant apoptosis when pretreated with calpeptin. Semiconfluent PC3 cells (5 × 10^4^/well) were treated with a range of concentrations of DTX and MTX in the presence or absence of CP (45 min; 20 μM) and assessed for apoptosis with ViaCount by flow cytometry after 48 h. CP pretreatment significantly abrogated DTX-induced apoptosis (**A,C**) and further limited DTX-mediated inhibition of proliferation (**B,C**) as well as MTX- and DTX + MTX- mediated apoptosis (**D,E**). Data presented are mean ± S.E.M. from three independent experiments performed in triplicate.

When MV release was inhibited in cells by pre-treatmetn with CP, thereby allowing intracellular levels of DTX and MTX to accumulate, significant cell death was observed, and proliferation was noticeably reduced, even with cells given reduced doses of drug (Fig. 4A–C, respectively). To confirm that MV release was occuring as a result of DTX/MTX-induced apoptosis and that this was inhibitable with CP, we found CP to more than halve numbers of MVs released from PC3 cells upon stimulation with DTX- (10 nM) (Supplementary Fig. S4A). We further identified a typical forward/side scatter tapering distribution dot plot by flow cytometry, with high levels of annexin V labeling (Supplementary Fig. S4B). NTA analysis showed a wide size distribution of MVs, peaking at 250 nm in diameter (Supplementary Fig. S4C) and electron microscopy revealed a characteristic morphology (Supplementary Fig. S4D). DTX/MTX combination therapy induced slightly more apoptosis after 48 h, compared to monotherapy (comparing Fig. 4E with A and D), and the addition of CP resulted in much higher apoptosis (~65% as opposed to ~25%) at 48 h with 10 nM DTX. These findings were repeated with one more androgen independent cell line, DU-145 (like PC3) and an androgen sensitive one, LnCaP (Supplementary Fig. S5).

### In a xenograft model of prostate cancer, inhibition of MV release with CP decreased tumor volume, intratumoral vascularization and cell proliferation whilst enhancing levels of apoptosis

**Figure 5:**
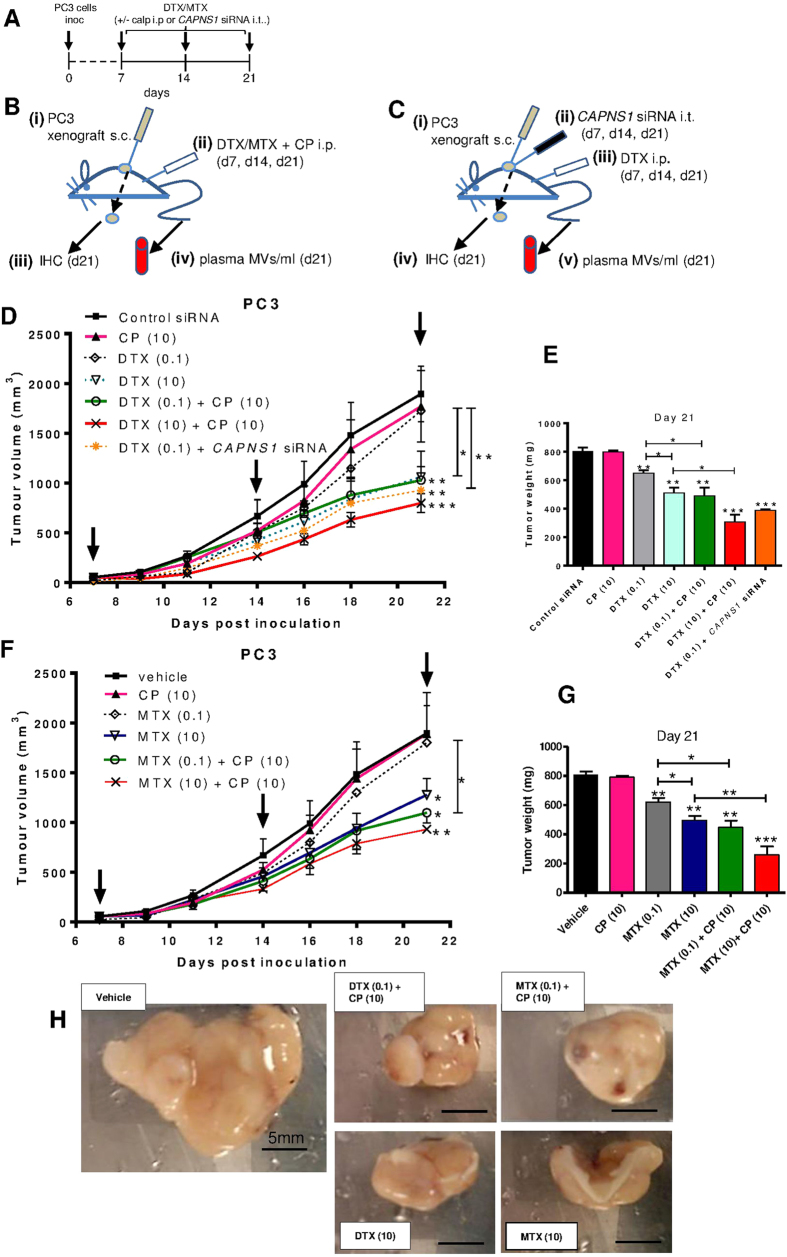
Mouse intraperitoneal xenograft model of prostate cancer shows enhanced reduction of tumor volume when chemotherapy is combined with pharmacological inhibition or siRNA gene silencing of calpain, to reduce MV release. The flow chart schedule (**A**) and experimental details and analyses carried out (**B,C**) resulted in tumor volume measurements (**D,F**) and terminal tumor weights (**E,G**) showing enhanced reductions following chemotherapy in the presence of CP. **H**, images of excised PC3 xenograft tumors in nude mice; Bar in ‘vehicle’ image, 5 mm and 6 mm in rest.

In the xenograft PCa mouse model, 7d after intraperitoneal injection of PC3 cells, and once tumors had become palpable, treatment was commenced according to the schedule in Fig. 5A. Mice were either treated with DTX or MTX together with CP intraperitoneally (i.p.) (Fig. 5B) or with intratumoral (i.t.) co-injections of *CAPNS1* siRNA (Fig. 5C). After three weekly treatments, immediately following euthanasia, resected tumors were subjected to IHC, and plasma MV levels determined.

Treatment with 10 mg/kg CP allowed a 100-fold lower dose of both DTX (Fig. 5D) and MTX (Fig. 5F) that could still bring about significant reductions in tumor volumes (Fig. 5D,F) as well as tumor weights (Fig. 5E,G), after 3 weeks. Tumor volumes (Fig. 5D) and weights on excision (Fig. 5E) were also reduced, when mice were injected weekly with siRNA targeting *CAPNS1* in combination with 0.1 mg/kg DTX. Plasma MV levels of CP-treated mice showed a concomitant small decrease (Supplementary Figure S6A) and body weight did not fall during the 3-week course of the experiment (Supplementary Fig. 6B), indicating no systemic toxic side effects. To gain an insight into MV levels in the xenograft tumor, we had additionally made single-cell suspensions from excised xenograft tumors from two mice given CP versus control to measure MV release from the cells. Although the results inferred reduced MVs/g tumor following CP administration, i.p., the procedure was considered unreliable.

**Figure 6:**
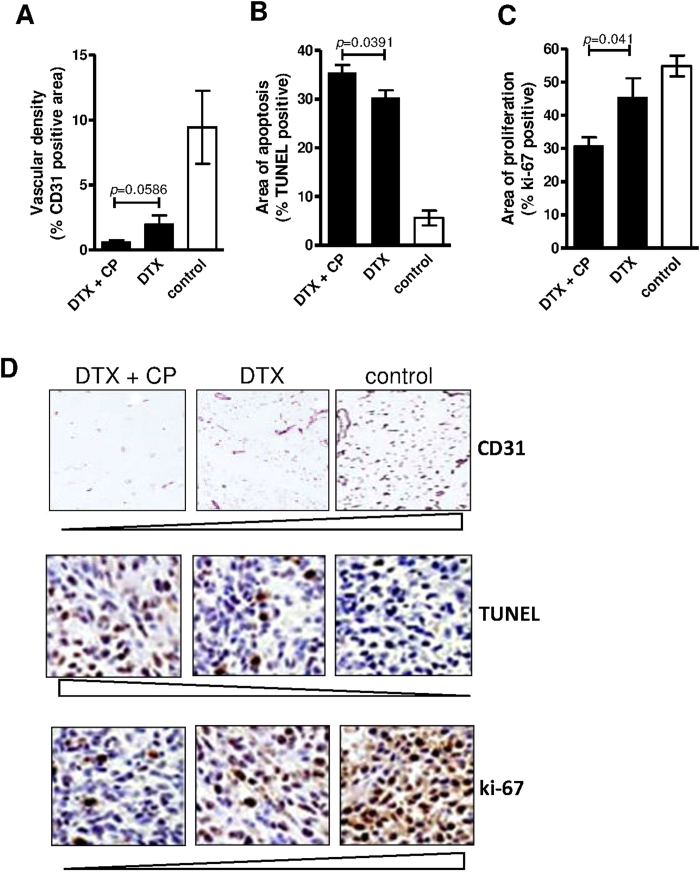
Pharmacological inhibition of calpain enhances DTX-mediated effects on angiogenesis, cell proliferation and apoptosis. Calpain inhibition with 10 mg/kg CP, i.p., enhances DTX-mediated inhibition of angiogenesis (10 mg.kg, i.p.) compared to DTX alone (10 mg/kg) as seen by decreases in vascular density (**A,D**) (% mouse CD31 (or PECAM-1) positive cells, blood vessels being identified as open lumens expressing at least one CD31^+^ cell), increases in apoptosis (**B**) (% TUNEL-positive cells) and decreases in numbers of proliferating cells (**C**) (% Ki-67 positive cells) in resected PC3 tumors, representative immunohistochemical staining respectively shown in (**D**).

DTX, besides being a chemotherapeutic drug used in PCa is also anti-angiogenic^29^. As expected, DTX treatment (10 mg/kg) itself decreased vascularization in tumor tissues as assessed by the surface area per field scoring positive (Fig. 6A,D) and this reduction was enhanced in the presence of CP (10 mg/kg). Thus although the reduction of xenografted PC3 tumor growth could be partly due to the decreased vascularisation, apoptosis and proliferation levels needed to be considered in the resected tumors.

A TUNEL assay further confirmed apoptosis in PC3 tumors collected after 3 weeks of DTX treatment (10 mg/kg) having induced a six-fold increase in the number of TUNEL-positive apoptotic cells compared with control and which was enhanced a further 6% with CP (Fig. 6B,D). We also found a significant inhibition of proliferation (% of Ki-67 positive cells) (Fig. 6C,D) and that CP (10 mg/kg) further reduced the levels of proliferating tumor cells, treated with DTX, by 15% (*p* < 0.05).

In summary, the *in vivo* model showed that the reduced PC3 tumor growth following combined treatment with DTX and CP which was likely to be the result of decreased intratumoral vascularisation, decreased proliferation and enhanced apoptosis of PC3 cells, had been potentiated by inhibiting microvesiculation of tumor cells, using CP.

## Discussion

Release of MVs carrying apoptotic enzymes, such as caspase-3, from cells undergoing intracellular stress, occurs as a defence against apoptosis^23^. As we noted recently in a review on the role of MVs in drug resistance^30^, inhibiting MV release allows caspase-3 levels to accumulate within stressed cells such that they succumb to apoptosis. Our review also alluded to another study which suggested that MVs laden with chemotherapeutic drugs may be released from cells resistant to chemotherapy^31^. The involvement of both P-gp and MRP1, as efflux transporters in MDR, as mentioned earlier first suggested a ‘non-genetic’ mechanism involving MVs transferring P-gp from MDR leukemic cells to drug-sensitive target cells^6^. These findings led us to explore a new hypothesis that MVs released upon treatment of cancer cells with chemotherapeutic agents can shed such drugs, and importantly to address whether inhibition of such MV release could ameliorate MDR in cancer.

**Figure 7:**
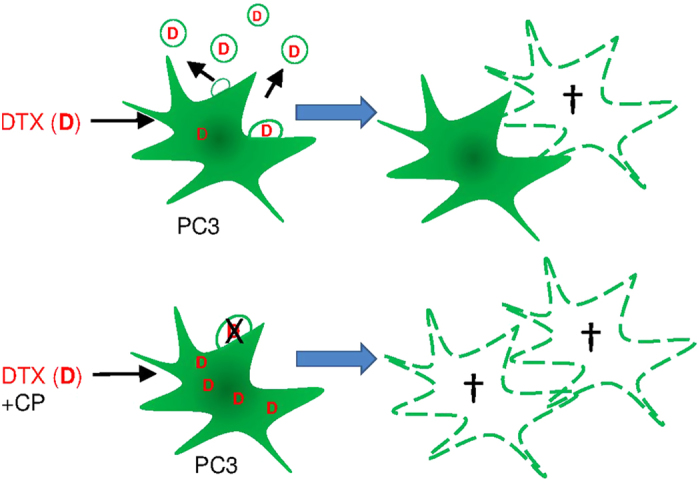
Schematic model for sensitizing cancer cells to chemotherapy by inhibition of MV release. Preventing removal of chemotherapeutic drug results in increased concentrations of intracellular chemotherapeutic agent(s), in turn leading to increased apoptosis.

The antimetabolite drugs MTX (and 5-FU) have been implicated in MDR and importantly the treatment of cancer patients with MTX (or DTX) leads to significant side effects due to the higher doses used. Here we show that these drugs when administered with CP could be given at doses up to 100 times lower and still induce effective killing of target PCa cells. This suggests a number of strategies that may be important for cancer therapeutics and indeed other diseases where cells may shed drugs through MVs. Specifically, we report a novel mechanism involving cancer cell expulsion of anticancer drugs via the release of MVs. We show for the first time that inhibition of this release through pretreatment of PCa PC3 cells with the membrane-permeant calpain inhibitor, CP, or by silencing the calpain gene, results in elevated levels of intracellular DTX, as had been previously suggested might happen for cytotoxic drugs in tumor cells treated with inhibitors of MV biogenesis^32^ and we showed recently with a novel inhibitor of microvesiculation^33^. This achieves a sensitization of cancer cells to DTX- (and MTX-) mediated apoptosis (Fig. 7). The capacity to inhibit MV release and to sensitize cells to chemotherapy is likely to also have an opposing effect by inhibiting removal of efflux transporters on individual cells but even that may not be important in view of the simultaneous limitation of MV-mediated transfer of such transporters to other cells.

In the process of MV release, activation of calpain, a Ca^2+^-dependent cysteine protease, able to cleave cytoskeletal actin filaments, may be instrumental. As well as CP sensitising cancer cells to chemotherapy by inhibiting MV release, we were aware that CP is a rho kinase activator capable of inhibiting calpains involved in apoptosis and cell cycle progression. By inhibiting apoptosis^34^,^35^, CP might by contrast modulate the effectiveness of chemotherapy *in vivo*. However, as we showed in our study CP had an overall effect of enhancing drug-induced early apoptosis, even *in vivo*. Indeed, in work on cancer therapies against breast cancer, combination therapy of calpeptin with histone deacetylase inhibitors was found to be beneficial^36^, and paradoxically calpain is often pro-apoptotic, cleaving and activating several apoptosis promoting substrates such as p53^37^,^38^,^39^,^40^.

Besides its role in microvesiculation, the calpains are an important family of proteases in cancer, whose abnormal expression has been implicated in various cancers, and which may be a factor in determining sensitivity to cancer therapy^41^; indeed m-calpain (calpain II) cDNA expression was increased by 20% in localized and 40% in metastatic prostate tumors compared to benign prostate and may be implicated in tumorigenesis^42^.

In this study we have inhibited MV release to prevent cancer cells shedding chemotherapeutic drugs. Both *in vitro* and in a xenograft model of PCa, 20–100-fold lower concentrations of DTX given together with CP matched the reductions in tumor volume achieved with DTX alone. In this study CP barely induced apoptosis. That in combination with DTX, apoptosis levels were increased means that the enhancing effect observed is not likely to be due to synergy and that some other mechanism must explain the observations; further supporting this, the same effect was observed with MTX, a chemotherapeutic drug with a different mechanism of action. We believe, whilst noting that at other concentrations, CP may indeed induce apoptosis, that, the increased sensitivity to DTX must be due to the increased dose of DTX available to the cell.

Although this strategy to enhance the levels of delivered drugs to diseased cells is very promising and may have wider applications in drug delivery, it still needs to be refined. New calpain inhibitors are being developed with higher specific activities^43^ which would need to be assessed. During the course of our work on MVs, we and others have used a host of inhibitors, including calpeptin, and methyl-β-cyclodextrin, as well as glyburide (unpublished), to abrogate MV release^44^. We are currently determining the specificity of proposed microvesiculation inhibitors and testing new ones, but perhaps alongside this, and for the same reason, inhibition of exosome release (exocytosis), should also be considered as an approach to tackle chemotherapy resistance.

## Additional Information

**How to cite this article**: Jorfi, S. *et al*. Inhibition of microvesiculation sensitizes prostate cancer cells to chemotherapy and reduces docetaxel dose required to limit tumor growth *in vivo*. *Sci. Rep*. **5**, 13006; doi: 10.1038/srep13006 (2015).

## Supplementary Material

Supplementary Information
