# Supplementary material for: Inhibition of microvesiculation sensitizes prostate cancer cells to chemotherapy and reduces docetaxel dose required to limit tumor growth in vivo
Source: Sci Rep. 2015 Aug 25;5:13006. doi: 10.1038/srep13006 (PMC4548233; doi:10.1038/srep13006)
Supplement: Supplementary Information [file srep13006-s1.pdf]

Inhibition of microvesiculation sensitizes prostate cancer cells to chemotherapy and reduces docetaxel dose required to limit tumor growth *in vivo*

Samireh Jorfi[a], Ephraim A. Ansa-Addo[a,b], Sharad Kholia[a,], Dan Stratton[a], Shaunelle Valley[a], Sigrun Lange[d,*], Jameel Inal[a,*]

[a]Cellular and Molecular Immunology Research Centre, School of Human Sciences, London Metropolitan University, U.K.

[b]Current Address: Department of Immunobiology and Cancer Immunology, Hollings Cancer Center, Medical University of South Carolina, Charleston, South Carolina 29425, USA.

[d]University College London School of Pharmacy, 29-39 Brunswick Square, London WC1N 1AX, U.K.

*co-corresponding authors: Tel: +44 20 7133 2122 or +44 20 7753 5895
E-mail address: j.inal@londonmet.ac.uk or sigrun.lange@ucl.ac.uk

Supplementary Figures

Supplementary Fig. 1 Calpain silencing in PC3 reduces CAPNS1 expression. Semi-confluent PC3 cells were transfected (or not) with 50 nM of control (Neg Cont) siRNA or various *CAPNS1* siRNA sequences targeting different regions for 48 h. Flow cytometry analysis of CAPNS1 knockdown in siRNA-treated cells showed that siRNA-#6 had the most significant reduction of CAPNS1 expression. To confirm siRNA-#6 for silencing calpain, PC3 cells were transfected with siRNA-#6 (50 nM) for 48 h. Relative CAPNS1 expression assessed by flow cytometry showed a 42% reduction compared to negative control siRNA. Data presented is the mean ± S.E.M. of three separate experiments performed in triplicate. ***P < 0.001; **P < 0.01; *P < 0.05 were considered statistically significant.

Supplementary Fig. 2 Representative HPLC chromatographs of MVs from untreated, control PC3 cells (A), cells treated with 100 µM MTX and of MTX standards, 2-100 µM (C).

Supplementary Fig. 3 Control HPLC chromatographs showing solvent peaks (A) and consistency of wavelength scan with MTX spectra from the literature, showing UV detection peak at 303 nm, (B).

Supplementary Fig. 4 Calpeptin reduces MV release from DTX-treated human prostatic cancer cells, PC3. Cells treated with 10 nM DTX, in the presence of 20 µM CP, released significantly fewer MVs. Released MVs were isolated, and identified as MVs from their typical scatter plot, high phosphotidylserine exposition (95% AnnexinV-positive), size estimation by nanosight (250 nm diameter) and from their characteristic morphology and size (~250 nm diameter) by transmission electron microscopy.

Supplementary Fig. 5 Prostate cancer cell lines LnCaP and Du-145, pretreated with calpeptin are sensitized to DTX treatment. Cells pretreated with CP (45 min; 20 μM) were treated with a range of DTX concentrations from 0.1 to 100 nM and after 48h were found to show higher levels of apoptosis where microvesiculation was inhibited.

Supplementary Fig. 6 Plasma MV levels at euthanasia and mouse body weights over the study. Plasma MV levels at euthanasia in the subcutaneous PC3 xenograft mouse model are diminished in animals treated with DTX and CP (both at 10 mg/kg), i.p., compared to animals given DTX alone. Mouse body weights throughout the experiment to eliminate toxicity effects due to treatment (B). Additional representative excised xenografted PC3 tumors (C) showing vehicle treated mice versus CP (10 mg/kg) and DTX + CP (both at 10 mg/kg); Bar, 4mm.

Supplementary Figures

Figure S1

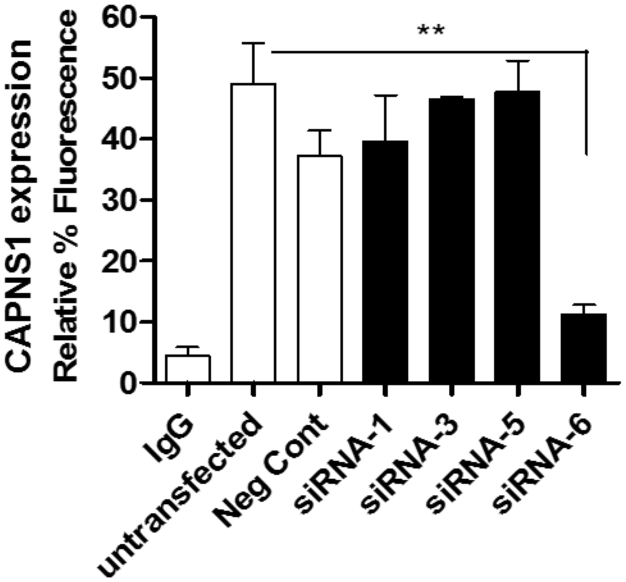

siRNA#1, 5' -CAC CTG AAT GAG CAT CTC TAT -3';
siRNA#3, 5' -AAG GTG GCA GGC CAT ATA CAA -3';
siRNA#5, 5' -CAG CGC CAC AGA ACT CAT GAA -3';
siRNA#6, 5' -TCC GAC GCT ACT CAG ATG AAA -3'.
Negative control siRNA, 5' -AAT TCT CCG AAC GTG TCA CGT -3'

Figure S2

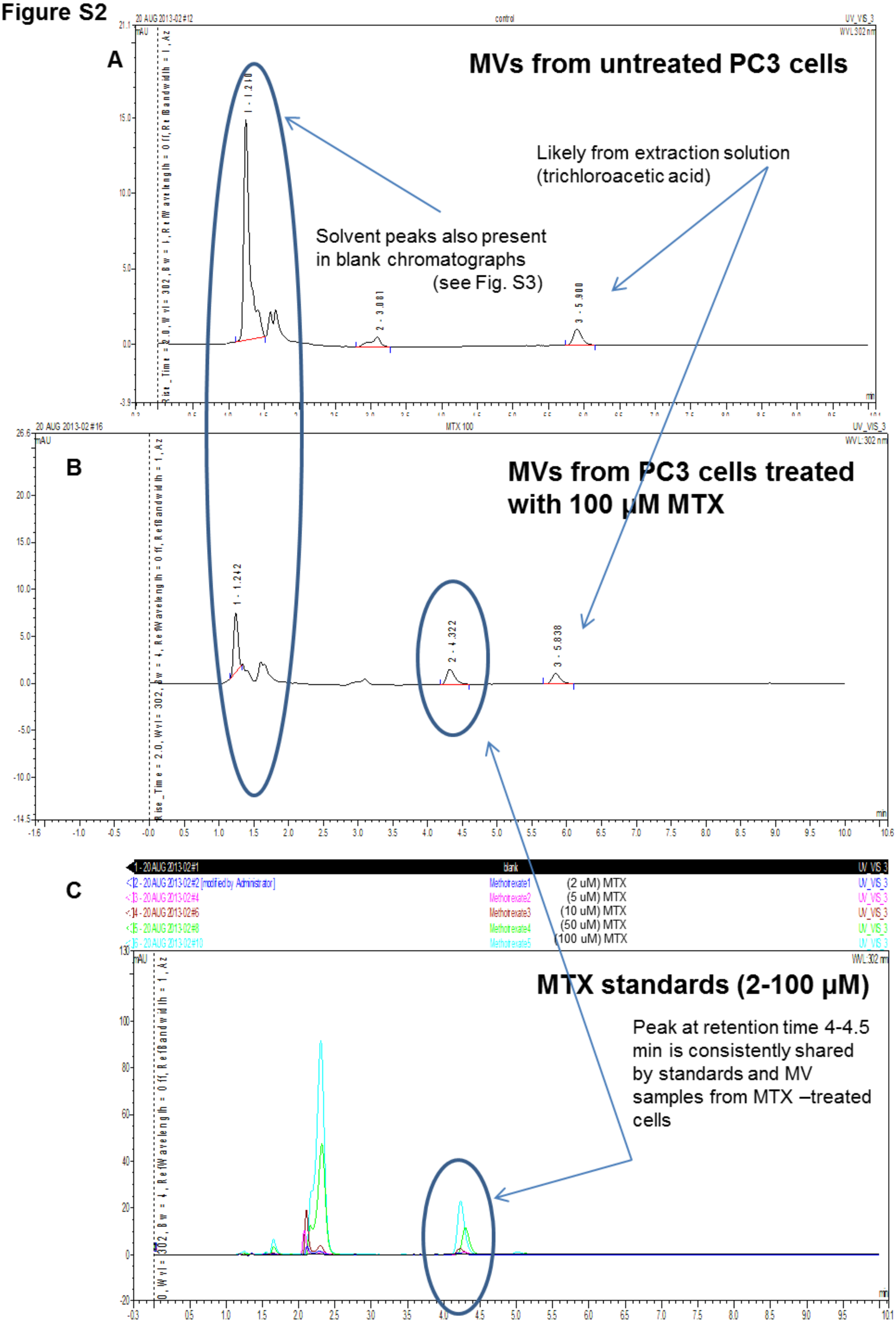
A
MVs from untreated PC3 cells
Likely from extraction solution
(trichloroacetic acid)
Solvent peaks also present
in blank chromatographs
(see Fig. S3)
B
MVs from PC3 cells treated
with 100 µM MTX
C
(2 uM) MTX
(5 uM) MTX
(10 uM) MTX
(50 uM) MTX
(100 uM) MTX
MTX standards (2-100 µM)
Peak at retention time 4-4.5
min is consistently shared
by standards and MV
samples from MTX –treated
cells

Figure S3

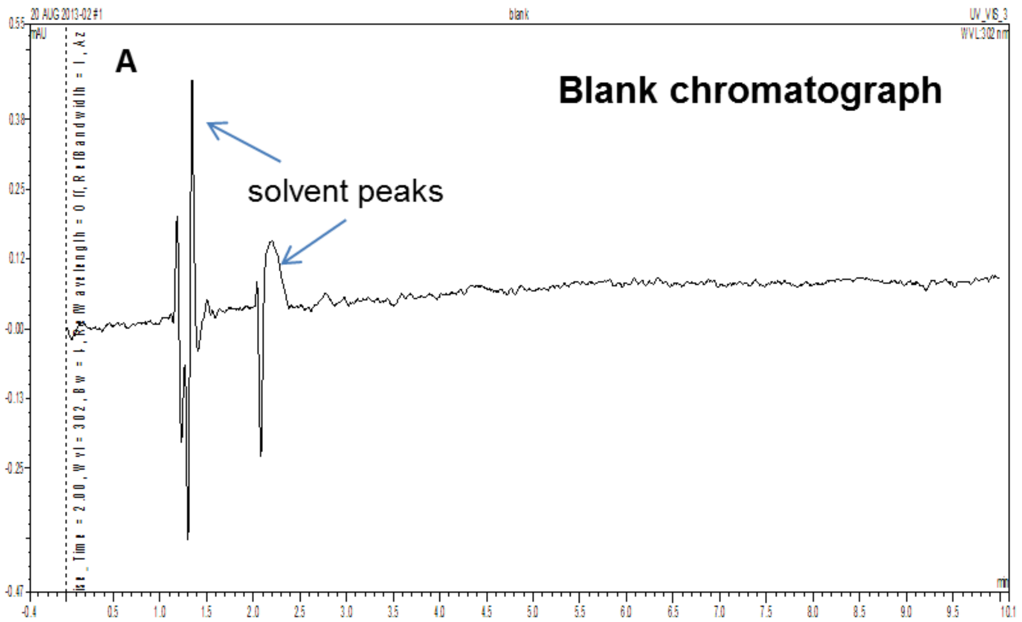
20 AUG 2013-02 #1
blank
UV_VIS_3
WVL:302 nm
mAU
A
Blank chromatograph
solvent peaks
min

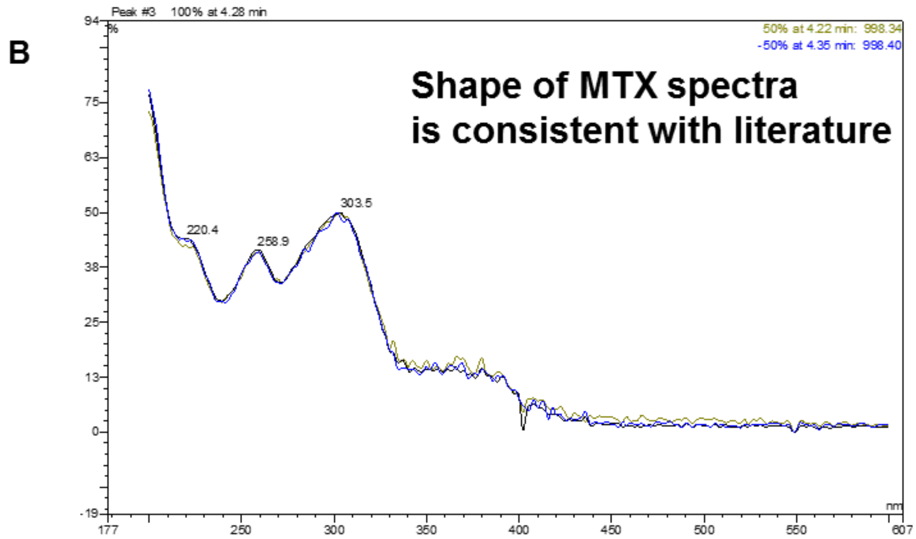
B
Peak #3 100% at 4.28 min
50% at 4.22 min: 998.34
-50% at 4.35 min: 998.40
%
Shape of MTX spectra
is consistent with literature
220.4
258.9
303.5
nm

Figure S4

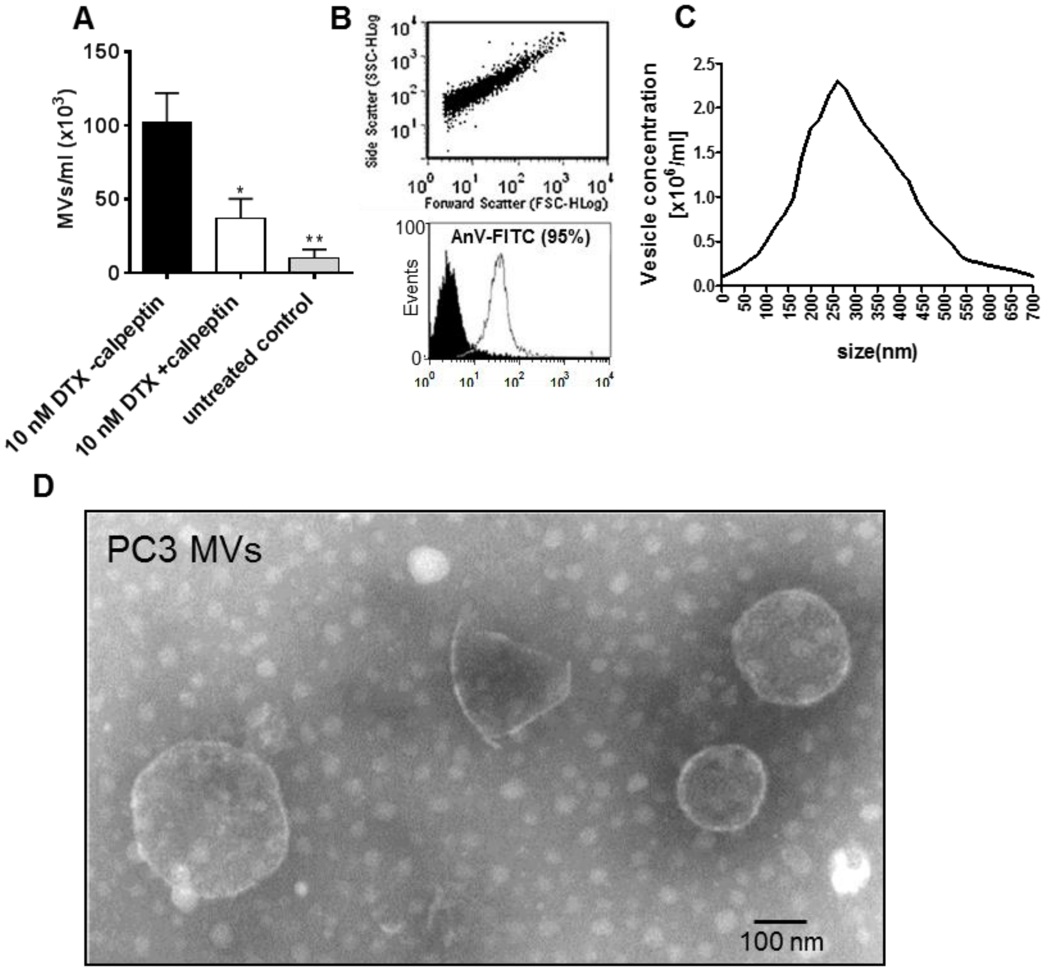
A
MVs/ml (x10³)
150
100
50
0
*
**
10 nM DTX -calpeptin
10 nM DTX +calpeptin
untreated control
B
Side Scatter (SSC-HLog)
Forward Scatter (FSC-HLog)
AnV-FITC (95%)
Events
C
Vesicle concentration [x10⁶/ml]
size(nm)
D
PC3 MVs
100 nm

Figure S5

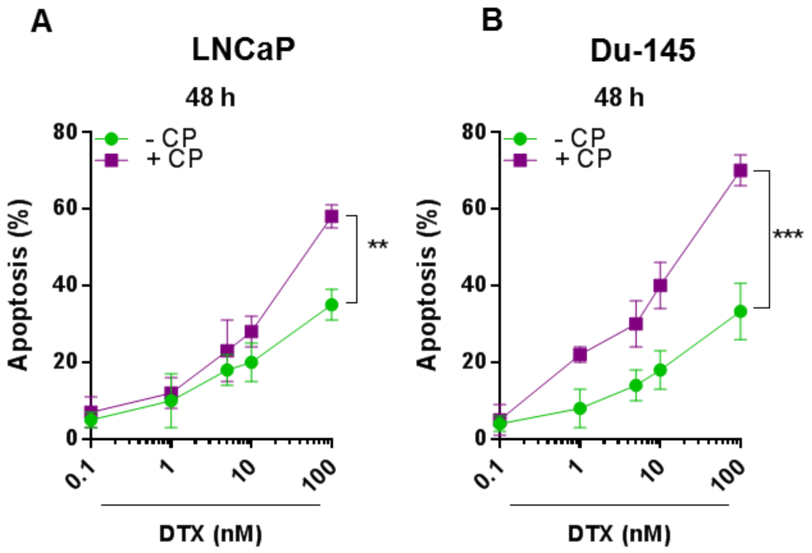

A
LNCaP
48 h
80
60
40
20
0
- CP
+ CP
**
Apoptosis (%)
0.1
1
10
100
DTX (nM)
B
Du-145
48 h
80
60
40
20
0
- CP
+ CP
***
Apoptosis (%)
0.1
1
10
100
DTX (nM)

Figure S6

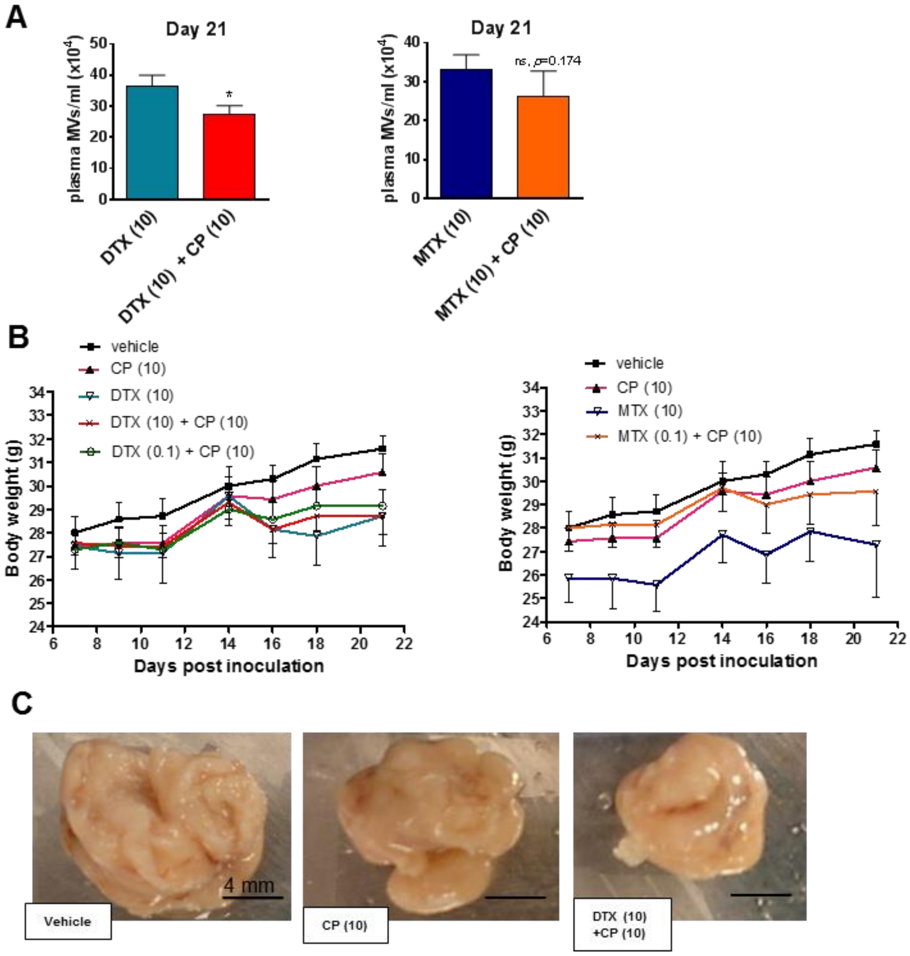

A
Day 21
plasma MVs/ml (x10^4)
50
40
30
20
10
0
*
DTX (10)
DTX (10) + CP (10)
Day 21
ns, p=0.174
plasma MVs/ml (x10^4)
40
30
20
10
0
MTX (10)
MTX (10) + CP (10)
B
vehicle
CP (10)
DTX (10)
DTX (10) + CP (10)
DTX (0.1) + CP (10)
Body weight (g)
34
33
32
31
30
29
28
27
26
25
24
6
8
10
12
14
16
18
20
22
Days post inoculation
vehicle
CP (10)
MTX (10)
MTX (0.1) + CP (10)
Body weight (g)
Days post inoculation
C
4 mm
Vehicle
CP (10)
DTX (10)
+CP (10)
